# Reference values of serum total IgE in Uppsala – comparison over four decades

**DOI:** 10.48101/ujms.v128.9892

**Published:** 2023-12-08

**Authors:** Robert Movérare, Eilif Persson, Andrei Malinovschi, Christer Janson

**Affiliations:** a Thermo Fisher Scientific, Uppsala, Sweden; b Department of Medical Sciences: Respiratory, Allergy and Sleep Research, Uppsala University, Uppsala, Sweden; c Department of Medical Sciences: Clinical Physiology, Uppsala University, Uppsala, Sweden

**Keywords:** Allergy, asthma, atopy, reference value, total IgE, upper limit of normal

## Abstract

**Background:**

Total immunoglobulin E (IgE) analysis is a common tool in allergy diagnosis. Suggested reference values for IgE are divergent and sometimes based on outdated assay methods. We aimed to validate the published reference values (geometric mean [GM]: 13.2 kU/L, upper limit of normal [ULN], 114 kU/L) shown in an Uppsala cohort from 1974 using Phadebas IgE PRIST, and the suggested clinical threshold of 100 kU/L (Zetterström and Johansson 1981).

**Methods:**

Immunoglobulin E was measured in two Uppsala cohorts from 1997 (Blood bank) and 2011 to 2013 (the European community respiratory health survey part III [ECRHS III]) using ImmunoCAP™ Total IgE. For the reference value calculations, exclusion criteria were atopy (both cohorts), doctor’s diagnosis of asthma and self-reported allergy (hay fever, rhinitis, rash) (only ECRHS III). Upper limit of normal was defined as mean + 2 standard deviations (SD) calculated using log-transformed values and back-transformation of the ULN prior to presentation. Common imputation methods for results below the assay range were evaluated.

**Results:**

The average GM was 14.2 kU/L (Blood bank, *n* = 63; imputation method range: 16.9–17.4 kU/L; ECRHS III, *n* = 113: 10.7–11.6 kU/L) and the overall mean ULN was 118 kU/L (Blood bank: 113–130 kU/L; ECRHS III: 104–128 kU/L). The clinical sensitivity and specificity of the 100 kU/L IgE threshold were 37.8 and 94.3% for atopy, 34.9 and 89.5% for doctor’s diagnosis of asthma, and 24.5 and 97.3% for any self-reported allergy (ECRHS III).

**Conclusion:**

The calculated ULN values were similar between the cohorts. We conclude that the total IgE reference values shown for Uppsala subjects from 1974 are still valid and suitable also for the ImmunoCAP Total IgE assay. The 100 kU/L threshold for total IgE had a low sensitivity but high specificity for atopy, asthma, and allergy.

## Introduction

Measurement of immunoglobulin E (IgE) in serum and plasma (generally known as total IgE) is commonly used as a complement to the investigation of sensitisation to allergens in patients with possible allergic disease. Total IgE is measured by laboratory assays, and is significantly elevated in patients with allergic asthma, hay fever or atopic eczema (1–3). However, there is major overlap in total IgE levels between atopic and non-atopic individuals (1, 4, 5), and a clear identification of atopic conditions will require further testing of the allergen sensitisation status (6). Total IgE may also be elevated in patients with immunodeficiencies and parasitic infections (7–9).

Phadebas IgE PRIST launched in 1976 was the leading total IgE assays in early attempts to establish reference values for IgE in healthy individuals (10). One of the most still cited studies is the one by Zetterström and Johansson from 1981 in which they reported a total IgE reference range using serum samples collected in 1974 from adult non-allergic individuals living in Uppsala and participating in a health survey (1). They found the geometric mean (GM) and upper limit of normal [ULN] of total IgE to be 13.2 kU/L and 114 kU/L, respectively. They also concluded that a total IgE concentration above 100 kU/L in patients with allergy-like symptoms was a strong indication for an atopic disease. Thus, IgE levels above 100 kU/L has since then commonly been used as a definition for atopy in clinical studies as well as an aid to diagnose allergy in clinical practice (11).

It has been argued that the reference range and the clinical threshold for total IgE recommended by Zetterström and Johansson is no more valid since their study is both old and performed with an assay not presently available. Indeed, more recent studies using the ImmunoCAP Total IgE assay have shown various values for the ULN, mostly at higher IgE levels (4, 5, 12). However, the definition of healthy subjects was not always as strict as in the Zetterström and Johansson study. Furthermore, genetic differences and environmental exposure may influence the IgE concentrations (5).

The aim of the present study was to validate the reference values reported by Zetterström and Johansson in 1981 by measuring IgE in two Uppsala cohorts collected in 1997 and 2011 to 2013 using the ImmunoCAP Total IgE assay. Moreover, the performance of the suggested and commonly used diagnostic threshold for total IgE at 100 kU/L was evaluated.

## Materials and methods

### Samples

Two sample cohorts from Uppsala were included. Venous serum samples were collected and prepared according to standard routines at the Uppsala University Hospital and were stored frozen (-20°C) until analysis. Besides total IgE measurement, all the samples were also analysed for the presence of IgE antibodies to common inhalant allergens. The first cohort from 1997 consisted of 95 serum samples from anonymous regular adult blood donors obtained from the blood bank of the Uppsala University Hospital (Blood bank cohort). They were considered as being healthy according to the routines of the blood bank, although having allergy without present symptoms were allowed for blood donors. The second cohort from 2011 to 2013 was from the European community respiratory health survey part III (ECRHS III) in Uppsala including 321 serum samples from adult subjects aged 40–66 years (ECRHS III cohort). Extensive clinical information was available for the subjects in the latter cohort based on self-reported questionnaire data and clinical examination. The ECRHS III study has been described more in detail elsewhere (13).

For the reference value determination, only non-atopic subjects, defined as being non-sensitised to common inhalant allergens (negative Phadiatop test), and/or subjects with negative questionnaire data (only available for the ECRHS III cohort) for having a history of doctor’s diagnosis of asthma or any current allergic symptoms (hay fever, rhinitis, rash) were selected. Other potential causes of elevated serum IgE, for example parasitosis, were disregarded because of the very low incidence in the geographic area.

### Total IgE

Serum IgE was measured using the ImmunoCAP™ Total IgE assay with the measuring range of 2–5,000 kU/L (Thermo Fisher Scientific, Uppsala, Sweden). The Blood bank cohort was analysed at Pharmacia Diagnostics in Uppsala in 1997, and the ECRHS III cohort was analysed at Academic Medical Centre, University of Amsterdam, The Netherlands, in 2013–14.

### Phadiatop

Serum IgE antibodies to a mix of common inhalant allergens were measured using ImmunoCAP Phadiatop™ (Thermo Fisher Scientific). Atopy was defined as having a positive Phadiatop test result (>0.35 Phadia arbitrary units per litre). The Phadiatop testing was performed at Pharmacia Diagnostics/Thermo Fisher Scientific, ImmunoDiagnostics in 1997 (Blood bank cohort) and 2019 (ECRHS III cohort).

### Statistics

Geometric mean of total IgE and the lower limit normal (LLN) and ULN were calculated according to Zetterström and Johansson (1). LLN and ULN were defined as the mean ± 2 standard deviations (SD), with mean and SD calculated on log-transformed values. Back-transformation of the LLN and ULN were done prior to presentation. No information is given in that paper about how results below the limit of quantitation (LOQ) of the assay were handled statistically. The arbitrary values given for values below the limit of quantitation (BLQ) will affect the SD and hence the ULN. In the present study, three common alternative methods for arbitrary value imputation for BLQ results (<2 kU/L) were evaluated. Below the limit of quantitation results were given an arbitrary concentrations equal to either: 1) LOQ, 2) LOQ/(square root of 2), or 3) LOQ/2 (14). Outlier sensitivity analysis of the study cohorts was performed using the Grubbs’ test.

Diagnostic performance of total IgE was evaluated from receiver operating characteristics (ROC) analysis where the area under curve (AUC) indicated the overall diagnostic performance in relation to the clinical parameter. The ROC curve was used to calculate the optimal IgE cut-off for each clinical parameter and was defined as the concentration corresponding to shortest distance from the ROC curve to the upper left corner. The diagnostic sensitivity and specificity of the established 100 kU/L total IgE threshold and the optimal IgE cut-offs were calculated.

Statistical calculations were done using Prism v.8.1.2 (GraphPad Software, San Diego, CA, USA).

### Ethics

The use of the ECRHS III cohort was covered by ethical approvals by the Ethic Committee at the Medical Faculty of Uppsala University, Sweden (Dnr 1999/313 and 2010/068). Since the samples in the Blood bank cohort were from fully anonymous regular blood donors at the hospital (no tracking possible to subjects), no ethical approval was deemed required for the use of the samples in the present study in accordance with Swedish regulations.

## Results

### Reference values for serum total IgE in Uppsala

A total of 63 samples (66.3%) from the Blood bank cohort showed negative Phadiatop results and were included in the study as samples from non-atopic apparently healthy blood donors (median total IgE: 18.3 kU/L, range: <2 – 177 kU/L, no outliers were indicated). The GM of total IgE of these blood donors was 17.4 kU/L and the ULN was 113 kU/L when using the BLQ 1 method for arbitrary value imputation of results below the LOQ of the assay (i.e. 2 kU/L was used for the BLQ results). The GM slightly decreased, and the ULN increased up to 130 kU/L, when using the BLQ 3 method (imputation with LLOQ/2 = 1 kU/L) because of an increased SD (Table 1).

**Table 1:** Reference values for total serum IgE in non-atopic and apparently healthy adult subjects from Uppsala 1974 to 2013, shown as geometric mean and lower and upper limit of normal. The results from 1974 was originally published by Zetterström and Johansson in 1981 (1). Three alternative methods were used for imputation of values below limit of quantitation of the ImmunoCAP Total IgE assay (BLQ 1–3).

Cohort (year)	*n*	Age (mean with range)	Total IgE assay	GM (kU/L)	LLN (kU/L)	ULN (kU/L)
Health survey reference group (1974)	175	46 years (17–85 years)	Phadebas IgE PRIST			
BLQ unknown				13.2	1.53	114
Blood bank cohort (1997)	63	Adults	ImmunoCAP total IgE			
BLQ 1				17.4	2.70	113
BLQ 2				17.2	2.44	121
BLQ 3				16.9	2.20	130
ECRHS III cohort (2011–2013)	113	54 years (41–66 years)	ImmunoCAP total IgE			
BLQ 1				11.6	1.28	104
BLQ 2				11.1	1.07	115
BLQ 3				10.7	0.89	128

Lower (LLN) and upper limit of normal (ULN) were calculated as the mean ± 2 standard deviations from log-transformed IgE concentrations and back-transformed prior to presentation. Results below the limit of quantitation (LOQ) of ImmunoCAP Total IgE (< 2 kU/L; Blood bank cohort, *n* = 3; ECRHS III cohort, *n* = 13) were given the following alternative arbitrary values for statistical calculations: BLQ 1 = LOQ; BLQ 2 = LOQ/√2; BLQ 3 = LOQ/2. BLQ stands for values below LOQ. *Other abbreviations:* n, number; y, years.

In the ECRHS III cohort, 113 subjects (35.2%) were classified as being healthy non-atopic subjects (50% females) and were included in the reference value calculations (median total IgE: 11.8 kU/L, range: <2 – 117 kU/L, no outliers were indicated). The GM of total serum IgE in this cohort was 10.7–11.6 kU/L dependent on which of the BLQ methods that was used (Table 1). No difference was seen between males and females (data not shown). Since as many as 13 samples (11.5%) had IgE <2 kU/L (LOQ), the choice of BLQ method for giving them an arbitrary value had a major effect on the reference range calculations. By using the BLQ 2 method, an ULN of 115 kU/L was obtained (Table 1).

The overall GM and overall mean ULN obtained from the Blood bank cohort and ECRHS III cohort using the three BLQ methods were 14.2 kU/L and 118 kU/L, respectively.

### Diagnostic performance of the 100 kU/L total IgE threshold and calculated optimal cut-offs

Having clinical information about allergic diseases in the ECRHS III Uppsala study, we studied the diagnostic performance of the commonly used 100 kU/L threshold for total IgE. The following three clinical parameters were studied: 1) Doctor’s diagnosis of asthma, 2) Any reported allergy (doctor’s diagnosis of asthma, symptoms of hay fever, rhinitis and/or rash), and 3) Atopy.

About 35% of the subjects with asthma had total IgE >100 kU/L, and the clinical specificity of that threshold was 89.5% (Table 2). The sensitivity of the IgE threshold for any reported allergy was lower (24.5%), but the specificity was high (97.3%). Also for atopy, the 100 kU/L threshold for total IgE showed a high specificity but the sensitivity was only 37.8% (Table 2).

**Table 2:** Diagnostic performance of total IgE for diagnosis of various clinical parameters in the ECRHS III Uppsala study (*n* = 321).

	Doctor’s diagnosis of asthma	Any reported allergy^a^	Atopy^b^
Affected subjects	83 (25.9%)	208 (64.8%)	111 (34.6%)
AUC (95% CI)	0.705 (0.641–0.770)	0.712 (0.656–0.769)	0.810 (0.763–0.858)
Common threshold	100 kU/L	100 kU/L	100 kU/L
Sensitivity	34.9%	24.5%	37.8%
Specificity	89.5%	97.3%	94.3%
Optimal cut-off^c^	31.0 kU/L	17.0 kU/L	26.5 kU/L
Sensitivity	61.4%	65.9%	73.0%
Specificity	69.3%	67.3%	74.8%

a Doctor’s diagnosis of asthma, or symptoms of hay fever, rhinitis, or rash.

b Positive Phadiatop test result (> 0.35 Phadia arbitrary units per litre).

c The total IgE threshold with shortest distance to the top left corner of the receiver operating characteristic curve.

ROC analysis showed that total IgE as diagnostic marker performed best in identifying atopy with an AUC of 0.810 (Table 2). The optimal cut-offs for the studied clinical parameters varied between 17 and 31 kU/L, and the corresponding sensitivity and specificity values did not exceed 70% for doctor’s diagnosis of asthma or any reported allergy-like symptoms.

## Discussion

In the present study, reference values for total IgE in non-allergic apparently healthy subjects from Uppsala were established in two separate cohorts collected in 1997 (Blood bank cohort) and 2011 to 2013 (ECRHS III cohort). They were compared with the reference values obtained in Uppsala samples collected in 1974 and analysed using Phadebas IgE PRIST and reported by Zetterström and Johansson in 1981 (1). The present study confirms that the GM of total IgE of 13.2 kU/L and the ULN of 114 kU/L published in 1981 may be considered as still being valid. The old study included a cohort of 175 characterised subjects (mean age: 46 years, range: 17–85 years) that had been carefully examined to exclude individuals with any signs of allergic disease involving tests for atopy. Similar criteria were used in the present study, in which all samples were analysed with ImmunoCAP Total IgE, a commonly used assay today in many laboratories.

The statistical method to calculate the reference interval in the present study followed the method used in the study by Zetterström and Johansson to enable direct comparison (1). A problem was how to handle values below the LOQ of the assay. The arbitrary values given to results BLQ have a direct effect on the SD and hence both the ULN and LLN. Since no information about BLQ values was given in the Zetterström and Johansson study, we decided to evaluate three commonly imputation methods to handle this issue (14). The imputation methods slightly influenced the reference values. The preferred imputation method is dependent on the skewness of the data and the frequency of BLQ values, and is a statistical subject beyond the scope of this study (14, 15). However, the ULN values were similar between the Blood bank cohort and the ECRHS III cohort, and the overall GM (14.2 kU/L) and overall mean ULN (118 kU/L) in the present study were very similar as reported in the old Uppsala study with samples from 1974 (1).

Several studies aiming to establish total IgE reference values in different populations were performed in the 1970s and 1980s. It was driven by the aim to use total IgE as an aid in clinical diagnosis of allergy. Later, newer studies have been performed to get updated reference intervals based on modern total IgE assays. Many of them have showed higher IgE ULN values than the Zetterström and Johansson study from 1981. Martins et al. reported a reference interval for total IgE in healthy adults in a study from 2014 using ImmunoCAP Total IgE (12). The ULN of their US-based population was 214 kU/L, which is considerably higher than 114 kU/L. However, in that study, no analysis of the atopic status was done, and the authors admit that some individuals might have had an underlying allergic disease despite showing no symptoms at the time of blood collection. In a Dutch study from 2003, Kerkhof et al. reported a ULN based on the 95th percentile for total IgE at 140 kU/L for female and 162 kU/L for male aged 20–44 years, and 116 kU/L for female and 205 kU/L for male aged 45–70 years (4). Carosso et al. presented total IgE data from 10 Western European countries including 2,249 young adult non-atopic subjects of the ECRHS II study, and reported the 95th percentile as 148 kU/L for female and 169 kU/L for men that were non-smokers (5). Recently, a Norwegian study reported a reference value (95th percentile) of 302 kU/L for total IgE using the ImmunoCAP method (16). However, no exclusion of atopic subjects was done which probably is a reason for the high ULN.

Hence, the total IgE reference values reported in later years, using modern automated total IgE assays, are generally diverse and higher than the reference values reported by Zetterström and Johansson in 1981. An advantage of calculating total IgE ULN based on mean plus 2 SD using log-transformed values compared to the 95th or 97.5th percentiles, as used in the cited studies, is that the former method is less dependent on few exceptional values that may have a major influence on the percentiles in small study populations.

Our present study is of value since it validates the ULN for total IgE established in 1981 and it indicates that no major changes of the IgE concentrations in healthy subjects have occurred in Sweden since the 1970s.

The present study also indicates that the suggested total IgE threshold of 100 kU/L as being predictive for allergy (atopic disease) is valid also today. In the ECRHS III Uppsala study, the 100 kU/L threshold for total IgE showed a high clinical specificity for atopy (positive Phadiatop test), doctor’s diagnosis of asthma, and any reported allergy-like symptoms, ranging from 89.5 to 97.3%. The sensitivity was considerably lower and did never exceed 40%. Lowering the threshold for total IgE to the individually calculated optimal cut-offs led to a sensitivity and specificity of 73.0 and 74.8% for atopy; but for doctor’s diagnosis of asthma and any allergy-like symptoms, the diagnostic accuracy was lower. Today it is generally recognised that measuring specific IgE antibodies has a higher diagnostic value than measuring total IgE in allergy considering the low diagnostic accuracy of the latter (17).

A limitation of the present study is the long time between the original investigation by Zetterström and Johansson published in 1981, in which the Phadebas PRIST IgE assay was used, and the present study, where all analyses were done using ImmunoCAP. However, the comparison of the results from the two total IgE assays and over time was supported by the common calibration of the assays against international reference preparations for IgE (18, 19). Besides the statistical method used to define the normal range of total IgE in healthy individuals, atopy, age, sex, smoking status and other factors can affect IgE levels, and hence the ULN values (5, 20). Only to a minor part were these factors considered in the present study because of lack of information. But similar to the study from 1981, both IgE sensitisation to common allergens and the presence of allergy-like symptoms (ECRHS III cohort) were used as exclusion criteria before the calculation of the reference values in an attempt to make a fair comparison between the studies. The actual sex distribution in the reference group from 1974 was not stated in the original study, but no differences in the total IgE levels between male and females, or between age groups, were observed (1). Neither did we observe any influence of the sex in the ECRHS III cohort. The fact that the Blood bank and ECRHS III cohorts in the present study were collected 15 years apart and also partly differed regarding inclusion criteria because of lack of allergy symptom information in the former cohort is a weakness. On the other hand, the similar results from both cohorts indicate no obvious trend for changing ULN values over time, and that the inclusion requirement of being non-sensitised to common inhalant allergens has a major impact on the ULN values. The relative low number of subjects included in our study cohorts decrease the statistical power and is of course another limitation. However, the aim was not to study the impact of various parameters like age and sex on serum total IgE. Others have shown that males tend to have higher serum IgE than females (4, 5), and that total IgE in adults decreases with age (21). It should also be noticed that serum IgE levels may differ between countries (22), and that good laboratory practice recommends that each laboratory establishes its own expected range of values or at least verifies reference intervals defined by others (23).

To summarise, the present study has validated the GM (13.2 kU/L) and ULN (114 kU/L) for total IgE reported by Zetterström and Johansson in 1981 for adult non-atopic apparently healthy individuals in Uppsala in a cohort from 1974. The present study showed an overall mean ULN of 118 kU/L for total IgE in serum from Uppsala subjects collected in 1997 and 2011 to 2013, and analysed using ImmunoCAP Total IgE. The previously suggested diagnostic threshold for total IgE (100 kU/L) as an aid in diagnosis of allergy was also evaluated and showed a high clinical specificity (89.5–97.3%) but a low sensitivity (<40%).
